# Designing a Future eHealth Service for Posthospitalization Self-management Support in Long-term Illness: Qualitative Interview Study

**DOI:** 10.2196/39391

**Published:** 2023-02-06

**Authors:** Hege Wathne, Ingvild Margreta Morken, Marianne Storm, Anne Marie Lunde Husebø

**Affiliations:** 1 Department of Public Health University of Stavanger Faculty of Health Sciences Stavanger Norway; 2 Department of Quality and Health Technologies University of Stavanger Stavanger Norway; 3 Research Group for Health and Nursing Sciences Stavanger University Hospital Stavanger Norway; 4 Faculty of Health Sciences and Social Care Molde University College Molde Norway

**Keywords:** colorectal cancer, eHealth service, heart failure, noncommunicable diseases, self-management, qualitative research, mobile phone

## Abstract

**Background:**

For patients with noncommunicable diseases (NCDs; eg, heart failure [HF] and colorectal cancer [CRC]), eHealth interventions could meet their posthospital discharge needs and strengthen their ability to self-manage. However, inconclusive evidence exists regarding how to design eHealth services to meet the complex needs of patients. To foster patient acceptability and ensure the successful development and implementation of eHealth solutions, it is beneficial to include different stakeholders (ie, patients and health care professionals) in the design and development phase of such services. The involvement of different stakeholders could contribute to ensuring feasible, acceptable, and usable solutions and that eHealth services are developed in response to users’ supportive care needs when transitioning to home after hospitalization. This study is the first step of a larger complex intervention study aimed at meeting the postdischarge needs of 2 NCD populations.

**Objective:**

This study aimed to explore the perspectives of patients with HF and CRC and health care professionals on patient self-management needs following hospital discharge and investigate how a future nurse-assisted eHealth service could be best designed to foster patient acceptability, support self-management, and smooth the transition from hospital to home.

**Methods:**

A qualitative, explorative, and descriptive approach was used. We conducted 38 semistructured interviews with 10 patients with HF, 9 patients surgically treated for CRC with curative intent, 6 registered nurses recruited as nurse navigators of a planned eHealth service, and 13 general practitioners experienced in HF and CRC treatment and follow-up care. Patients were recruited conveniently from HF and CRC outpatient clinics, and the nurses were recruited from the cardiology and gastro-surgical departments at a university hospital in the southwest of Norway. The general practitioners were recruited from primary care in surrounding municipalities. Semistructured interview guides were used for data collection, and the data were analyzed using thematic analysis.

**Results:**

In total, 3 main themes were derived from the data analysis: expecting information, reassurance, and guidance when using eHealth for HF and CRC self-management; expecting eHealth to be comprehensible, supportive, and knowledge promoting; and recognizing both the advantages and disadvantages of eHealth for HF and CRC self-management. The data generated from this interview study depicted the diverse needs for self-management support of patients with CRC and HF after hospital discharge. In addition, valuable suggestions were identified regarding the design and content of the eHealth service. However, participants described both possible advantages and disadvantages of a remote eHealth service.

**Conclusions:**

This study is the first step in the development of an eHealth service for posthospitalization self-management support for long-term illnesses. It concerns patients’ supportive care needs and user requirements of an eHealth service. The findings of this study may add value to the planning and development of eHealth interventions for patients with NCDs.

## Introduction

### Background

Noncommunicable diseases (NCDs) are defined as diseases or conditions that tend to be of long duration and slow progression [1]. NCDs are estimated to be responsible for >70% of all deaths (41 million people) per annum worldwide, and the most common NCDs that account for the most deaths are cardiovascular disease and cancer [2]. A growing number of patients with NCDs such as heart failure (HF) and colorectal cancer (CRC) are prone to comorbidities, a high rate of readmissions, and complex health care needs [3]. Similar to all long-term chronic conditions, patients may require day-to-day self-management [2], which may result in increased treatment burden (ie, patient work) [3,4]. They also experience an ongoing need for a trustworthy contact with the health care system that can deliver qualitatively sound health-related information [4,5].

HF is a progressive and complex clinical syndrome with a tremendous symptom burden, including dyspnea, fatigue, edema, and sleeping difficulties [6,7]. It is associated with periods of acute deterioration and an increased risk of hospitalization [8,9]. CRC is one of the most prevalent cancers in the world [10]. Owing to improvements in health care systems, the number of survivors of this cancer has increased [11]. This causes patients with CRC to live with the illness for a longer period, similar to patients with other chronic diseases [12].

### The Importance of Posthospitalization Self-management

The period following hospital discharge is deemed particularly vulnerable for many patients as they transition from care in a safe hospital setting to individual self-care at home [13]. Moreover, many struggle to perform recommended self-care and navigate the health care system, particularly when posthospitalization care is poorly executed because of inadequate coordination of resources or follow-up from home health care interventions or general practitioners (GPs) [4,9].

Self-management may be defined as the strategies that individuals undertake to promote health, manage an illness, and manage life with an illness [14]. Self-management is increasingly recognized as a fundamental component of NCD care as adequate self-management skills may help patients with NCDs control their chronic conditions [15]. However, self-management demands a substantial effort from the patient, requiring routine work and timely adjustment of therapy to avoid exacerbation events and facilitate detection and avoidance of recurrence and prevention of disease progression. In addition, patients must solve practical problems, manage physical and psychosocial consequences and lifestyle changes, and know when and how to seek appropriate medical advice [16-18]. In HF management, self-care is a cornerstone as it improves treatment effectiveness and reduces hospital admissions. However, many patients with HF have a limited understanding of the basic elements of the nature of HF; they often misinterpret HF symptoms and feel inadequately informed [19]. Consequently, patients with HF are often unprepared to take charge of their self-management tasks after hospital discharge [19]. Therefore, self-management interventions that promote and support self-care after hospital discharge are becoming increasingly important for this group of patients [19]. In patients with CRC, a decrease in postoperative length of stay has been observed [20]. However, many patients are likely to experience changes during the initial postoperative phase, including changes in bowel habits, pain, fatigue, mobilization, dietary challenges, and physical and psychological distress (ie, anxiety and depression) [21]. Many patients with cancer also experience ongoing difficulties in assessing support and services at home [22]. Hence, the transition from active treatment in the hospital to self-care at home is a period when patients with CRC most feel insecure and require intervention [12].

### eHealth and Current Self-management Programs

Today, health care systems worldwide are faced with the challenge of managing care for long-term chronic illnesses [23]. An extraordinary and promising resource that promotes chronic disease management, including patient self-management, is eHealth [24,25]. eHealth is defined as *the delivery of health care using modern electronic information and communication technologies when health care providers and patients are not directly in contact and their interaction is mediated by electronic means* [25]. The purpose of eHealth is to change patients’ behavior and improve their health status [26]. eHealth may also enhance treatment durability as patients can receive support and reinforcement of skills after hospitalization during the transition phase from hospital to home [27]. Research suggests that patients with chronic illnesses supported by innovative eHealth solutions within a care pathway feel more motivated to engage in self-management behavior [28,29]. Furthermore, a study investigating video consultation as an alternative to face-to-face consultation among patients with CRC and their treating surgeons showed that video consultation is equivalent to face-to-face follow-up consultations in terms of patient satisfaction and perceived quality of care [30]. This may suggest that the quality of patient-provider interaction can be maintained using digital solutions [31]. However, self-management support interventions need to be tailored to the individual and their specific condition and context [32]. Moreover, the growing number of patients with NCDs requires a more dynamic and flexible follow-up approach, and eHealth support may be a beneficial strategy to meet the posthospital discharge needs of patients with NCDs [3,24].

### Fostering Patients’ eHealth Acceptance

The potential benefits of eHealth have been widely described. However, the use of eHealth remains low, and evidence on how to design eHealth services to meet the complex needs of patients is inconclusive [33]. An explanation for the lack of results is that, during the development process, insufficient attention is paid to the needs, wishes, and context of the prospective end users [32]. The credibility, value, and success of eHealth lie in its ability to demonstrate positive outcome effects, where end users’ engagement in the design and development of eHealth services is important to overcome adaptability barriers [34]. Today, several studies have highlighted the lack of user involvement in the development of such interventions [35-38]. To foster the successful use of eHealth interventions, it is important to develop eHealth interventions in response to users’ needs rather than as a technological innovation [37], and for self-management support to be effective, it must be provided by suitable health care professionals (HCPs) [39]. In particular, nurses are important to support self-management as enabling patients to understand and cope with their disease, its treatment, and its consequences is a core competence of nursing [39]. Nurses can, through remote digital care, guide and support patients in self-care by providing them with analytic skills to interpret bodily signals and by activating them to take the appropriate measures to prevent exacerbation events [40]. Therefore, nurses may play a pivotal role in fostering patient acceptance and support and guiding patients toward sustainable and effective self-management [40].

### Current Knowledge Gap and the Need for This Study

Research on eHealth-based support interventions for people with NCDs recommends that the interventions be theory-based and hold an element of communication in addition to web-based material [41]. eHealth programs are found to be most efficient when led by multidisciplinary teams where HCPs can encourage the patients to adhere to the program and when the eHealth program is designed based on the outcomes to be achieved [42]. Critical gaps remain in the design and evaluation of self-management interventions, with a lack of patient and clinician involvement [43]. Rochat et al [44] emphasized the importance of iterative involvement of end users in the design and evaluation process of a coaching solution to support the postdischarge needs of patients with HF. Furthermore, the results of Fairbrother et al [45] showed that telemonitoring enhanced patients’ knowledge and understanding of their condition but that further work is required by patients and professionals to develop a shared understanding of self-management and the role and function of telemonitoring as an enabling intervention within this context.

Although appearing different in terms of diagnosis, treatment, and prospects, patients with HF and patients surgically treated for CRC both represent conditions in need of long-term follow-up care, necessitating extensive self-management capacity and skills in the transition to home after hospital discharge [46,47]. Moreover, the 2 patient groups have the most vulnerable types of NCDs and may serve as proxies for the broader NCD field. Self-management interventions across different chronic conditions can contribute to improved health outcomes [48]. A recent study found that survivors of CRC were positive toward postdischarge monitoring and follow-up. The participants especially requested features for information, questions and answers regarding nutrition and weight, and provision of social support [30]. Research on digital self-management interventions for patients with HF has shown varied results [43,49]. When used for posthospitalization follow-up, eHealth interventions can positively affect quality of life, whereas their impact is less evident for self-care and readmissions [49]. However, research on how patients with NCDs can best be supported in self-management during transitions is sparse, including which eHealth-based support interventions are best suited for follow-up care [13]. To many patients with HF or CRC, the transition to self-management after hospital discharge represents a void of professional health care that may leave them unprepared for self-managing these tasks at home [50,51]. Thus, bridging the gap in health care between hospital discharge and home by developing more seamless eHealth services from inpatient to outpatient care supported by hospital assistance seems necessary if patients with NCDs are to achieve adequate self-care and feel safe [6,16].

### Aims of This Study

In this study, which is the first step of a larger complex intervention aimed at developing and testing a generic eHealth service for patients with NCDs, the aims were twofold: (1) to explore the supportive care needs of patients with HF and patients surgically treated for CRC in transition to home and (2) to identify different stakeholders’ (ie, patients, registered nurses [RNs], and GPs) views on important content and functions of a future eHealth service designed to meet patients’ supportive care needs in the transition from hospital discharge to home. The research questions were as follows: (1) What are the essential needs regarding self-management support among patients with HF and CRC transitioning from hospital to home that can be met by a future eHealth service? (2) How can a future eHealth service be best designed, and what are perceived to be essential content and functions to foster patient acceptability from the perspective of patients and HCPs?

## Methods

### Study Setting and Design

This study is part of a larger research project, *eHealth@Hospital-2-Home*, and includes three phases: (1) developing a nurse-assisted eHealth service, (2) assessing feasibility and piloting the service, and (3) carrying out a randomized controlled trial [3]. This study pertains to the main project’s first phase and will inform the modeling and adaption (ie, content and functions) of a future hospital-based, nurse-assisted eHealth service for patients living with HF or CRC. In this study, an exploratory and descriptive qualitative design was applied. Data were collected using semistructured interviews with patients with HF, patients surgically treated for CRC, RNs, and GPs to explore their perspectives on patients’ supportive needs following hospital discharge and how a future eHealth service can be best designed to foster patient acceptability.

### Study Population

The study’s patient populations comprised patients with HF and patients who had received surgical treatment for CRC with curative intent. The selection criteria for both patient groups were age between 18 and 80 years, attendance to an outpatient clinic at hospital A, ability to understand and speak Norwegian, capability to take part in the interview, and no acute medical crisis. The patients were recruited during a scheduled follow-up appointment at either the HF outpatient clinic or the gastro-surgical outpatient clinic. The study sample also comprised nurses and GPs. The nurses, engaged as nurse navigators (NNs) in the project, were RNs from 2 hospitals in the southern part of Norway (hospitals A and B) and experienced with HF or CRC treatment. In total, 4 of the RNs worked at hospital A: 2 in a medical intensive care unit in the cardiology department and 2 in a gastro-surgical ward. The final 2 RNs worked in an HF unit at hospital B. The GPs worked as part of primary care services in municipalities corresponding to hospital A, and they all had ≥2 years of experience as GPs. The GPs were invited to participate in the study because of their experience with various patient groups, including HF and CRC, after hospital discharge.

A total of 39 persons were approached, and 38 (97%) consented to participate in the study. Of these 38 participants, 10 (26%) were patients with HF, 9 (24%) were patients surgically treated for CRC, 6 (16%) were NNs, and 13 (34%) were GPs. The age of the patients with HF ranged from 49 to 78 years, and that of the patients with CRC ranged from 58 to 76 years. The RNs were all women and ranged in age from 26 to 37 years. Their work experience as RNs ranged from 3 to 11 years, and 67% (4/6) of the nurses were nurse specialists (ie, intensive care and stoma nurses). The GPs were aged 35 to 66 years, and their work experience as medical doctors was between 7 and 27 years. All but 23% (3/13) of the GPs were specialized in general medicine, as well as 15% (2/13) who were also specialized in community medicine. Please see Table 1 for an overview of participant demographics.

**Table 1:** Characteristics of the study sample (N=38).

Characteristic		Patients with CRC^a^ (n=9)		Patients with HF^b^ (n=10)	GPs^c^ (n=13)	NNs^d^ (n=6)
Age (years), range		58-74		49-73	35-66	26-34
**Sex, n (%)**						
	Male	3 (33)	7 (70)		9 (69)	0 (0)
	Female	6 (67)	3 (30)		4 (31)	6 (100)
**Educational status of patients, n (%)**						
	Primary school	3 (33)	0 (0)		N/A^e^	N/A
	High school	5 (56)	6 (60)		N/A	N/A
	College or university	1 (11)	4 (40)		N/A	N/A
**Work experience for GPs and NNs** **(years)** **, n (%)**						
	1-3	N/A	N/A		1 (8)	1 (17)
	4-7	N/A	N/A		2 (15)	4 (67)
	>10	N/A	N/A		10 (77)	1 (17)

^a^CRC: colorectal cancer.

^b^HF: heart failure.

^c^GP: general practitioner.

^d^NN: nurse navigator.

^e^N/A: not applicable.

### Recruitment

Participants were recruited from different settings and through various means. The patients were recruited conveniently [52] from 2 different outpatient clinics at hospital A: 1 HF clinic and 1 gastro-surgical clinic. They were contacted for participation by a designated recruitment nurse during a routine follow-up appointment. They received an information and consent letter from the recruitment nurse and gave their consent to be contacted by the researcher to receive further information and possibly schedule an interview. Of the 20 patients who agreed to be contacted by the research team, only 1 (5%) declined participation after reading the information letter and receiving further information about the study. The RNs were recruited as NNs on personal request by members of the research team or by the head nurse at the department. The GPs were encouraged to participate in the study after receiving general information about it at a meeting for GPs. In addition, they received a reminder by email and as a posting on a web page specifically aimed toward GPs in the area. Those who were willing to participate responded with an email to the researcher and provided their contact information. The researcher contacted the consenting participants and scheduled a suitable time for the interview. The GPs received a gift certificate (value of approximately €100 [US $108.30]) as compensation for the loss of work hours.

### Ethics Approval

Ethics approval for the study was obtained from the Norwegian Centre for Research Data (611713). However, ethics approval for the study was considered *not notifiable* by the Regional Committees for Medical and Health Research Ethics (169884). The research was conducted according to the Declaration of Helsinki, the Regional Committees for Medical and Health Research Ethics, and the research guidelines of the 2 university hospitals.

### Informed Consent

The participants were recruited voluntarily and received information about confidentiality, anonymity, and the right to withdraw from the study at any time [53]. Informed consent was obtained after the participants were given information about the nature of the study and aspects of participation. Data were anonymized and securely stored according to Norwegian Centre for Research Data guidelines.

### Data Collection

According to the study’s explorative and descriptive design, the aim was to seek new insights into specific issues and serve as a basis for further research [54]. Therefore, this research was conducted with a specific purpose: to inform the design and modeling of an eHealth intervention. This influenced the development of interview guides and data analysis. Semistructured interview guides were developed by the research team and were used to (1) explore the participants’ experiences with the transition phase from hospital to home and the specific needs of the patients during this period and (2) explore their views on the content and functions of a future digital health care solution. At the start of each interview, the patients were asked which digital tools they used daily (eg, smartphone, laptop, and iPad). They were then given a brief overview of the future eHealth solution and possible monitoring devices and asked if such a digital service was something they would be able to operate, either alone or with the help of family members. They were then asked to share their experiences of the period following hospital discharge and their first weeks at home. On the basis of these experiences, they were asked to share what they imagined would be helpful content and functions in a future eHealth posthospitalization follow-up service. The nurses and GPs were also given an overview of the future eHealth service as an introduction to the interviews. The answers from the participants were to form the basis of the content, components, and technical features of the eHealth solution. For a more detailed overview of the questions from the interview guides, please refer to Textbox 1.

The interviews were conducted by the first author both face-to-face and, because of COVID-19 restrictions, digitally through Zoom (Zoom Video Communications) and by phone. A total of 95% (18/19) of the patients were interviewed by phone, and 5% (1/19) were interviewed face-to-face in their home. Of the 6 NNs, 4 (67%) were interviewed face-to-face in an office at their workplace, and 2 (33%) were interviewed via Zoom because of travel restrictions. All the interviews with the GPs (13/13, 100%) were conducted over the phone. During the interviews, the interviewer used follow-up questions such as “What do you mean when you say...?” “Can you elaborate?” and “Is it correct of me to understand what you just said as...?” All the interviews were audio recorded.

**Examples of questions from the interview guides.**

- Patient questionnaire—questions to establish digital experience
  Do you use any digital tools daily (eg, smartphone, iPad, computer/laptop, or smartwatch/Fitbit)?
  Can you give me some examples of how and for what you use your digital tools?
  Do you ever use digital tools in connection with health, disease, or treatment?
- Patient questionnaire—questions to help shape the content in an eHealth service
  During your transition from hospital to home, what did you:
    experience as problematic?
    need more information about related to your condition or treatment?
    need the health care system to help you with regarding managing or complying with the medical regimens you were recommended?
    need in terms of emotional support?
  Do you have any thoughts on how the health care system could have offered you support after discharge?
- Patient questionnaire—questions to help shape the design and layout of an eHealth service
  In your opinion:
    What would be useful components in a postdischarge eHealth service (eg, illustrations, pictures, type of information, checklists, chat, video, notifications, and reminders)?
    For an eHealth service to be useful for you in your daily life, what would be important factors to consider?
    How would you prefer to interact/communicate with health care providers (HCPs) using an eHealth service (eg, chat, video consultations, or phone)?
  What is your experience with monitoring devices (eg, blood pressure, saturation, and weight), and which features seem useful in an eHealth service if you were to assess and monitor your own health condition?
- HCP questionnaire—questions to help shape the content of an eHealth service
  In your opinion/experience:
    What challenges do patients with heart failure (HF)/patients treated for colorectal cancer (CRC) face after hospital discharge?
    For patients to cope with long-term illness, what is important to prepare them for?
    Why and for what reasons do the patients with HF/CRC contact you after hospital discharge?
    What do you expect from patients after they are discharged from the hospital regarding self-management and adherence?
- HCP questionnaire—questions to help shape the design and layout of an eHealth service
  In your opinion/experience:
    Where do patients collect information if they have questions regarding their disease and course of treatment?
    What type of health information is suitable for an eHealth service?
    What should an eHealth service look like and what seem like core functions and content in such a solution?
  How do you imagine working with an eHealth service would affect your everyday work?

### Data Analysis

The audio recordings were transcribed verbatim by the first author and analyzed using a thematic analysis approach in a stepwise process in accordance with Braun and Clarke [55]. In the first step, the transcriptions were read and reread to form an opinion on the overall content and meaning. The fully transcribed interviews were distributed among all the authors, and the texts were subsequently marked and commented on. In the second step, the first author searched for sentences and longer units of text, analyzed them, organized them into possible meaning units, and gave them preliminary codes. In the third step, the meaning units were sorted further and placed into a coding scheme where similar codes were grouped into different categories with a focus on identifying variations, similarities, and differences within each category, aiming to form potential themes. During the third step, all the authors participated by commenting and making suggestions on the coding and categories. In the fourth step, each category was reviewed, refined, and grouped into more distinct subthemes. The subthemes were identified and named by characterizing content, and by framing differentiated concepts, preliminary main themes were also identified. This back-and-forth process continued until a consensus was reached between all the authors. In the fifth step, categories and subthemes were re-examined, regrouped, renamed, and placed within their correct main themes. In the sixth step, the final schemes were decided on and presented in tables consisting of meaning units, codes, categories, subthemes, and main themes. All the authors participated throughout the various steps of the analysis process to ensure trustworthiness. The coding was performed manually, and no software was used to structure the process.

The data were analyzed groupwise, starting with the transcribed interviews of the patients with HF before starting on the transcribed data material from the patients with CRC. These data were subsequently handled as the previous group, with the various coding schemes systematically compared for similarities, differences, and variations in patient experiences. As the preliminary coding showed similarities across the patient population, the preliminary codes and coding schemes were re-examined, regrouped, renamed, and placed into categories concurrently. Furthermore, as this study aimed to tailor a service to meet the follow-up needs of patients, the findings of the data material from the 2 patient groups formed the basis for the analysis of HCP data material. The stepwise data analysis is shown in Multimedia Appendices 1 to 4.

## Results

The findings provided valuable insights into three main themes: (1) expecting information, reassurance, and guidance when using eHealth for HF and CRC self-management; (2) expecting eHealth technology to be comprehensible, supportive, and knowledge promoting; and (3) recognizing both the advantages and disadvantages of eHealth for HF and CRC self-management. For a detailed overview of the main themes and their corresponding subthemes, codes, and data extracts, refer to Multimedia Appendices 1 to Multimedia Appendices 4.

### Expecting Information, Reassurance, and Guidance When Using eHealth for HF and CRC Self-management

The first main theme was supported by 2 subthemes: *a need for personalized information and advice about what to expect after discharge* and *a need for personal interaction to reduce postdischarge uncertainty and anxiety.* These 2 subthemes address the supportive care needs of the patients after hospital discharge.

#### A Need for Personalized Information and Advice About What to Expect After Discharge

The patients with HF described a variety of needs after hospital discharge, and they seemed to have an endless demand for information. Their information needs were mostly related to their diagnosis, the course of the disease, and symptom management. In the period following hospital discharge, many patients with HF described a lack of understanding of what HF was and how the disease would present itself:

I didn’t know I had heart failure. I thought it was a heart attack, not that it was called heart failure. I thought they had fixed me.

Patient with HF 1

Some patients with HF found it difficult to make individual decisions based on the information they had received, and many lacked confidence in handling their symptoms. Therefore, the patients emphasized that the information they received should be more tailored to fit their individual needs. A patient with HF expressed uncertainty concerning the information he had received:

I used to be allowed to drink 1.5 liters per day, and then they increased it to 2 liters. But if I forget, is that dangerous? Will I start retaining water again? And can I drink more when I exercise and sweat a lot? It would have been nice to know what dangers were associated with it because they say you should stick to what you’re told.

Patient with HF 7

Many patients with HF described the period after discharge as chaotic. From living a normal life, many were discharged to a life in which they had to pay attention to a disease that they knew little about and take precautions by adjusting to taking several new medications every day. They lacked knowledge about their illness and found it difficult to understand and manage. Moreover, many of the RNs and GPs emphasized that giving information to patients with HF was particularly difficult as the information had to cover a range of different aspects of their lives:

A person with heart failure has so many questions. Some existential, like: Why did this happen to me? But also: How can I live? What can I do? How much can I push myself? Is it dangerous to have sex? Can I go to the store? It is a dramatic and once-in-a-lifetime experience that happens to them, and they have so many questions.

GP 4

The information needs of patients with CRC were less related to their diagnosis than those of patients with HF. After their tumor was surgically removed, their need for information was mostly dominated by postoperative issues such as bowel function, pain, infection, and leakage, with bowel function causing the most concern. Some of them were also unprepared for the postsurgical pain and the duration of the pain, as described by the following patient:

My bum—it was like barbed wire. It was sown and I had stitches for weeks...and the pain...it lasted for months. I didn’t know it would be like that when they removed my bowel.

Patient with CRC 9

Other patients with CRC reported that their physical condition returned to normal within the first few weeks following discharge and that their need for information decreased accordingly. Nevertheless, the GPs described unexpected postoperative complications as the main reason why patients surgically treated for CRC made contact after hospital discharge. In addition, some of the patients with CRC needed help with practical matters in the initial weeks spent at home:

Some get complications that may be problematic, but otherwise, they mostly need help with practical things, like sick leave, stoma equipment, or other practical things to help them get their lives back on track.

GP 4

Patients treated for CRC also expressed a need to be prepared for what may happen after discharge or “answers to the most common questions that arise after surgery,” as a patient with CRC phrased it. Some also had concerns about nutrition and activity level after returning home from the hospital:

The information I got from the hospital was that I could eat as normal and move around as much as my body allowed me to, but after the surgery, I couldn’t do as much as I wanted.

Patient with CRC 2

Although the 2 patient groups were different in terms of both diagnosis and which symptoms they needed to be aware of after discharge, some challenges were common between them. Most of the patients in both the CRC and HF groups stated that they wished they had been more prepared for how exhausted they would feel after hospital discharge:

I am a very impatient person, so I wanted to exercise the following day. But everything took longer than I thought, which was very frustrating for me because I thought I could just snap my fingers and all my problems would be solved.

Patient with HF 6

I thought it was fantastic to come home to my family and have them near me. However, I was very exhausted and tired.

Patient with CRC 7

The tiredness was described as worrying by the patients, especially as it affected their everyday chores and substantially limited their level of activity. Furthermore, many patients were accustomed to having well-functioning bodies before hospitalization and were not prepared to experience a reduced activity level after discharge. Several of the RNs and GPs recognized activity as an undercommunicated subject and emphasized that both patient groups should be made aware of the importance of restitution after they leave the hospital:

When they’re discharged, it’s not like they’re expected to be back to their normal selves. The convalescence continues. They must take their time and not wear themselves out because they have a belly that has been opened, and they have to consider the wound. But there’s the housework and the showering and all these everyday things...often small things, but not so small for the patients.

CRC nurse 1

#### A Need for Personal Interaction to Reduce Postdischarge Uncertainty and Anxiety

The second subtheme, *a need for personal interaction to reduce postdischarge uncertainty and anxiety,* emerged as a response to the many and various descriptions of the patients’ continuous need for psychosocial support after hospital discharge. Patients with HF described worries and uncertainty about their disease progress, both how long their heart would last and whether it would just suddenly stop. Many described the period after hospital discharge as especially uncertain and frightening, and a lack of information before discharge seemed to contribute to their anxiety and fear of dying:

I didn’t know what was going to happen. I was constantly afraid. No one called me to ask how I was, and I really missed that because I wasn’t even that old, and I thought I was going to die. Nobody told me anything.

Patient with HF 4

Furthermore, some patients with HF were overwhelmed by their many “self-management duties” after discharge. Many also described uncertainty about the future and struggled with existential worries and fear. Patients with CRC also had postdischarge worries and expressed a need to talk to someone after returning home from the hospital. They typically worried about cancer relapse or if the cancer had metastasized so that they would need chemotherapy after the surgery. The waiting period between having the surgery and receiving the histological result was described as particularly straining. In addition, the RNs referred to this as a time of uncertainty and anxiety for the patients:

I think they are more anxious after the surgery and up until they receive the histology result: that’s when they are scared. And also in regard to further treatment—if they have to do everything all over again or need radiation and chemotherapy.

CRC nurse 2

Both patient groups described a need to talk to someone after discharge, and many used family and friends for social support. However, disease-specific issues, symptoms, and advice that included how to conduct necessary changes in their everyday lives were subjects that they wanted to discuss with HCPs:

I would have liked to ask some questions to someone who knows. That we could have communicated a bit back and forth.

Patient with CRC 1

Furthermore, some patients wondered whether their reactions after discharge were normal and expressed a wish to communicate their situation to someone other than their family after they had returned home. In fact, participants from all groups suggested using the digital service to facilitate contact with other patients who had gone through the same thing. Peers were brought up as potential supporters with whom the patients could discuss their various experiences and feelings. Many of the patients sought confirmation from other patients—some sort of affirmation that their reactions and thoughts after discharge did not deviate too much from that of other newly discharged patients. Thus, in this context, peers were suggested as particularly useful supporters:

Of course, people are different, but there probably are some similarities as well. So, if you could meet others with similar experiences and ask like: what was your reaction to that? Maybe get some kind of confirmation that your thoughts and feelings are the same as everybody else’s.

Patient with HF 5

In addition to peers, links to various user organizations, support groups, and validated and reliable websites with scientifically correct information were proposed by both patients and HCPs as something that could potentially support patients.

### Expecting eHealth to Be Comprehensible, Supportive, and Knowledge Promoting

The second main theme was supported by the following 2 subthemes: *a need for a manageable and useful eHealth solution* and *a need for communication tools and sources for knowledge acquisition.*

#### A Need for a Manageable and Useful eHealth Solution

Strong agreement existed among all the participants that, if the digital solution was to be manageable and useful, its most important quality should be ease of use:

It has to be as easy to use as a phone. It can’t be difficult to access, then you just wouldn’t be bothered.

Patient with CRC 8

The participants explained ease of use as easy access, a logical and intuitive interface, clear and visual text, and understandable words and symbols. The solution also had to be beneficial for patients of various age groups:

I think it has to have an easy layout for it to work both for them at age 20 and for those at age 90. It has to be easy, with easy adjustable letters, and a front page with visual and easy things to click on. Not too advanced.

HF nurse 5

Furthermore, several of the participants in both the patient and HCP groups emphasized a need for reliable and easily accessible information within the solution. However, they thought that the information within the eHealth service should be formulated in a way that everyone could understand, including educated people and those without formal education. The information language should not be too complicated, and the medical terms and formulations should be simplified. One of the GPs accentuated the importance of more straightforward information:

I think a great deal of public information has a high level of learning. However, there should be a point to making the information comprehensible. You really shouldn’t create insecurity, but patients [with HF] need to know why they get breathless.

GP 12

This GP’s statement was confirmed by participants in both patient groups, stressing that the information within an eHealth service should not cause stress or discomfort. One should also avoid using words that may trigger unnecessary fear. One of the patients with HF was very specific in his advice:

You want it [the medication] to prevent early death. That’s what all the instructions say. But for an anxious person—I don’t think it’s wise that they read the words “early death” because that’s all they’ll see, if you follow? Maybe if it was rephrased to prevent an unfortunate development or bad result. Then they wouldn’t have to read the word death, right? Maybe then they wouldn’t get so anxious.

Patient with HF 1

Moreover, the HCPs were concerned with making the information of eHealth services explanatory and educational, ideally making the users more knowledgeable and capable of managing their specific disease, including possible precautions and lifestyle changes:

I think it’s important to think educational—to provide them with knowledge they can use long-term. Have I gained weight? Am I breathing more heavily if I walk these steps? And also, it’s wise for them [the patients] to base it on things that are close to them. Like the stairs in their own house or an uphill in their neighborhood. It will make it easier for them to measure.

GP 5

#### A Need for Communication Tools and Sources for Knowledge Acquisition

All the participants mentioned several tools and various sources that could promote knowledge and skills among the patients. They explained how regular contact with HCPs after discharge could provide patients with individual and more tailored information that may make them more receptive to changes in their condition and sensitive to the importance of responding to them. The possibility of keeping in touch with the health care services and sending questions and receiving answers from HCPs was suggested by many of the participants, with various ways of contact and communication promoted. Chat was considered to be the fastest and easiest way to connect and communicate. In addition to being time-effective, sending questions through a chat may feel less threatening than reaching out via video or telephone:

I like chat because it is fast, and I feel I can use the time I need to explain. I don’t feel like they are thinking: “she needs to hurry up.” I can take my time and still get answers.

Patient with CRC 8

Among the HCP population, video was found to be an appropriate and advantageous communication tool as it gave them the possibility to see the person they were talking to. This was confirmed by the patient participants, who emphasized the benefits of relating to a face rather than just to words. Chat was seen as an adequate communication tool for simple and straightforward questions and messages. However, if something needed to be assessed by an HCP, video seemed more trustworthy. By using video, the patient could show their surgical wound or stoma, or their breathing pattern or leg edemas could be assessed by qualified HCPs. One of the GPs also emphasized the following:

I would wish to talk to them. To see them and talk to them. They could show me things, like swollen ankles or something, and also I can get an impression how they breathe. Or if they’ve had a bowel operation...their wound or skin. You do get a better impression with video.

GP 11

Many participants in both the patient and HCP populations also suggested answering questions regularly, such as questionnaires or checklists, as something that may promote appropriate self-management activities and keep patients updated on their condition. Some of the HCP participants suggested that answering questions within the eHealth service could function as a reminder for the patients—regular cues that reminded the patients “to do what they’re supposed to do,” as one of the RNs phrased it. By logging on to a digital system and actively replying to questions regularly, patients, especially those with HF, thought that they would become more aware of their behavior and habits, as well as becoming more receptive and willing to engage in appropriate and health-promoting self-management activities. The idea of receiving feedback on checklists was also emphasized as particularly beneficial:

Checklists would be great, and blood pressure, follow-up regarding medication and maybe also weight, like: Have you gained weight? How is your weight? Have you retained water in your body? Do you have to increase your medication? Those are the sorts of things where you don’t know what to do, and then you could write like: I am feeling like this and that—what shall I do?

Patient with HF 9

However, it was emphasized by one of the GPs that the questions in the eHealth service had to be disease- and symptom-specific so it would be easy for the patient to connect it to their specific condition:

Take heart failure, for instance, if there was something you wanted to measure, you could ask: How many stairs can you climb? How many meters can you walk on a flat road? Your morning weight? That will give them something to compare and they can see changes. I’m very skeptical to “how are you” questions because that’s very subjective and not necessarily related to the condition.

GP 5

During the interviews, the participants were asked to share their thoughts and experiences regarding home monitoring and vital signs. This was a subject on which the participants had different opinions. Most participants, both patients and HCPs, had a positive attitude toward home monitoring. They proposed that, if the patients monitored their vitals at home after receiving proper training, it could help them gain a better overview of the disease progress and make them more attentive to symptoms and complications and be more in control of their health condition:

For most patients, I think it would feel very safe and reassuring to know that they have something concrete to pay attention to. I think it could be meaningful for them during the first period. I also think that saturation, weight, and blood pressure are familiar for most patients today. It may also give them more understanding and insight into their own disease.

HF nurse 6

Some of the participants also stated that home monitoring could form the basis for information and reduce the number of visits to the physician’s office. However, some of them were skeptical about home monitoring and claimed that leaving patients in charge of such measurements might be perceived as burdensome and potentially cause unnecessary worries for the patients, especially if the measurements showed discrepancies:

You can get a bit caught up in it [home monitoring] as well, and when you have gone through something like this, you’ll probably be monitored pretty good anyway, so I don’t know if it is such a good idea.

Patient with CRC 5

Nevertheless, although not every patient or GP saw the benefits of home monitoring, some patients with HF were used to taking various measurements, such as measuring their blood pressure, regularly counting their heart rate, or paying attention to their weight. Many of these patients, along with some of the GPs, proposed that it would be beneficial if the digital solution had graphical or statistical visualizations of the various measurements that the patients had taken along with feedback on the measurements if they were irregular:

I am keeping an eye on my weight, so maybe if I had the possibility to enter the numbers and see them as a graph. I think that would be interesting.

Patient with HF 2

### Recognizing Both Advantages and Disadvantages of eHealth Services for NCD Self-management

The last main theme comprised the following 2 subthemes: *recognizing eHealth as a tool for follow-up care* and *concerns about eHealth as a tool for follow-up care*.

#### Recognizing eHealth as a Tool for Follow-up Care

All the RNs were positive toward eHealth and argued that digital follow-up care would prolong the period in which patients were under supervision from health care services, which could strengthen the relationship between the patient and the hospital. However, they primarily argued that a digital follow-up service could be supportive for patients during the vulnerable phase following discharge:

I think it [an eHealth service] may serve as a connector between the patients and the hospital after discharge. The first few days after returning home are the most uncertain, so I think that every patient may benefit from being watched over by someone from the health care services who checks if they manage everyday life at home. And then they can rest knowing that they’re not all by themselves.

CRC nurse 1

RNs also emphasized that an extended follow-up period, which an eHealth service may provide, could offer the patients more adapted information. This could lead to an increased sense of security and make the patients and families more capable of managing life at home. In addition, a digital solution with symptom registration could lead to the early detection of changes and subsequently prevent readmissions. Patients with CRC also recognized potential benefits of digital follow-up care, especially in connection with postsurgical complications, as described by the following participant:

I think a digital solution to help people post discharge would be helpful for those with complications. If there was something wrong with the surgical wound for instance.

Patient with CRC 2

A positive attitude toward digital follow-up care was supported by most patients with HF. An eHealth service could be reassuring for patients after discharge and lower the threshold for asking questions. Furthermore, several of the patients with HF shared stories about how they felt insecure, lonely, or “left to themselves” after they came home from the hospital, and some were under the impression that a digital solution could have reduced some of the negative emotions they experienced after discharge:

When I got home after discharge, the house was freezing cold, and I was all alone. I felt really lonely. Coming home to a cold and empty house, without anybody around you...That’s what I remember as the worst part. So maybe if I had an iPad? Or access to a chat or something. Maybe I wouldn’t have felt so completely left alone.

Patient with HF 5

The GPs mostly viewed digitalization in health care as beneficial as a nurse-assisted eHealth service could potentially make health care services more approachable by simplifying communication and lowering the barrier to contact. They also suggested that maintaining contact through a digital solution may feel less threatening for the patients, as well as putting less strain on the health care system. This was a view shared by many of the nurses, especially the CRC nurses.

#### Concerns About eHealth as a Tool for Follow-up Care

Some of the GPs expressed concerns about this type of follow-up care. They seemed worried that a nurse-assisted eHealth service would disturb the patient-GP relationship. They recognized that the eHealth service could be a tool to help patients cope and give them answers to many of the questions they had after discharge, but a digital solution should never interfere with the interaction between them and their patients, as the following GP emphasized:

I think they need to be reminded to contact their GP so that they can get help to assess the situation or control things, because I think there are quite a few readmissions. So, it [eHealth] could be a smart way to reach people when they are in trouble and need help, but I don’t think it’s wise to let it replace the GPs’ evaluations.

GP 9

Some of the patients, mainly the patients with CRC, expressed skepticism about the need for a digital follow-up service. In total, 2 factors were highlighted as particularly challenging when it came to digitalization of the health care system, with the first being the human factor. Communicating and receiving follow-up care without physical contact or connection with an actual person was viewed by some of the patients as foreign and “cold.” One of the patients with CRC said the following:

Isn’t that just a complete waste? In my sense, it is much better to have contact with people over the phone or with your GP. You lose all contact. It is just a machine.

Patient CRC 4

However, the digital competence factor seemed to cause more concern for other patient participants. They indicated that eHealth and its technical features would be difficult for some people to understand, and they questioned whether everyone would have sufficient digital competence to operate the solution:

I think this digital solution is very appropriate. But I’m not sure that everyone will find it convenient to use. Some will not have the skills, and some will have a bit of an aversion to this computer world. Not everyone can use this type of equipment.

Patient with HF 2

A third issue regarding eHealth was highlighted by some of the patients with CRC: who would benefit from using an eHealth service? The patients with CRC seemed to believe that digital follow-up care was most appropriate for patients who experienced some type of surgical or medical complication. Many of the patients with CRC used the phrase “differently sick than me” to describe patients who would benefit from using an eHealth service after discharge. A patient with CRC said the following:

I think if I was different. Say I had metastasis. Then I would want to have contact, but as long as I felt well and they said that there wasn’t anything wrong...then I just would have wanted to go to my regular follow-ups. I think it would have been more burdensome if I had an app and felt that I had to write to someone. That would have taken up too much of my time.

Patient with CRC 6

“Differently sick” included everything from having surgical complications to having a stoma or being diagnosed with metastasis. The prevailing view of the patients with CRC was that, if the operation and postoperative course went without complications, there was no need for digital follow-up care. Having to deal with a digital solution after discharge was thought to add to the treatment burden rather than decrease it. These patients’ view of digital follow-up care stands in contrast to that of the CRC nurses, who claimed that newly discharged patients frequently contacted the hospital ward with various questions after CRC surgery. Answering phone calls from insecure and worried patients was described as work and resource-demanding. The CRC nurses spoke about how they expected the patients to understand the information they were given during hospitalization. In addition, they provided the patients with a discharge letter and expected them to collect the necessary information from there or call their GP with any additional questions, as described by the following nurse:

We often experience that patients call the hospital ward because they are insecure after discharge. Even though the discharge letter clearly says they should contact their GP. We get quite a few phone calls.

CRC nurse 2

## Discussion

### Principal Findings

This study applied a qualitative interview approach to explore various stakeholders’ perspectives on self-management needs after hospital discharge and investigate how a future eHealth service can be best designed to foster patient acceptance, support self-management, and ease the transition from hospital to home. We found that patients with both HF and CRC had unanswered questions and faced various challenges after hospital discharge. Some struggled to understand which self-management tasks were necessary and what precautions they should take to avoid complications or exacerbations. The statements from the patients regarding posthospitalization self-management challenges were confirmed and expanded upon by the HCPs. In addition, the participants shared many valuable opinions and ideas about the content and functions of a future eHealth service.

The first and overarching main theme demonstrated how additional information and follow-up care are necessary for patients during the transition to home after hospital discharge regardless of diagnosis. The patients in this study described common challenges in their daily lives, including fatigue; confusion regarding activity level; and psychosocial challenges such as negative thoughts, worries about the future, and a general need for more support. These findings are supported by existing literature describing challenges following discharge for patients with both cancer [22] and HF [56]. In addition to information, the patients in this study emphasized a need for more tailored advice about what to expect after discharge and personal interaction to reduce postdischarge uncertainty and anxiety. Tailored information and personal interaction are conditions that may be closely intertwined and should be seen in relation to each other as insufficient information or a lack of advice about disease management may lead to extensive worrying and a lack of confidence to engage in necessary self-management after hospital discharge [19]. Moreover, depression and anxiety may impede an individual’s ability to engage in self-management behaviors [57]. For many patients, especially those living with long-term illnesses, hospital discharge often marks the start of a new round of self-management activities [58]. Thus, the findings from this study highlight the importance of “equipping” patients with NCDs with more tailored knowledge and skills to reduce or prevent psychological conditions that may hinder self-management.

The second main theme captured the participants’ views and ideas on the design and technical functions of a digital solution as well as identifying relevant content that could meet the support needs of patients with HF and CRC during the transition phase from hospital to home. As the patient participants and GPs in this study described common challenges across the 2 patient groups, there seem to be various core functions that could shape the content of a digital platform. Easily accessible, understandable, and nonfrightening disease-specific information; multifaceted knowledge-enhancing functionalities; and different communication sources such as chat, video, checklists, and home-monitoring devices were the most prominent features suggested by the participants. According to Nymberg [59], many patients can see possibilities with the use of eHealth as an improvement, alternative, or complement to existing health care. However, there is a strong need for user-friendly and well-adjusted digital tools compatible with patients’ needs [31,59].

An important finding of this study is that being able to exchange messages and receiving informational support from HCPs would help reinforce the self-management skills of patients with NCDs and give them a better understanding of their medical condition with its accompanying symptoms and complications. In addition, receiving emotional support from HCPs after discharge was thought to help patients cope with their postdischarge worries and the need for practical advice. Evidence exists for the positive effects of eHealth on patients’ perceived support [39]. Support is essential to help individuals accomplish self-management tasks, and it is an important strategy to reduce the burden of chronic disease [60]. Moreover, from a patient perspective, acceptance of technology is greater when it is not perceived as replacing in-person care [61]. Therefore, it is important that eHealth services have a “human component” and serve as a complement to, not a replacement for, usual care [16,59]. In that sense, nurses may be an asset in future eHealth solutions for patients with NCDs as instigators of contact with them after hospital discharge. Regular contact and follow-up care from designated nurses may also contribute to fostering patient adherence to treatment [62].

The third main theme identified the participants’ views on eHealth in general and specifically on eHealth as a tool for self-management. The participants in this study had different opinions on the value of and need for eHealth. Although most participants in both the patient and HCP populations seemed curious and positive toward eHealth, some expressed skepticism. This is in line with other research showing that many patients have different perceptions and expectations of eHealth [63,64]. On the one hand, eHealth may be viewed as something difficult and troublesome, and on the other, it may be seen as something that makes things easier. In this study, the various perceptions of eHealth and digital follow-up care seemed to be related to human or technological factors. Participants most in favor of eHealth attributed this to the advantages of patients being able to contact and communicate with HCPs at the time of exacerbations or worries, thus receiving follow-up care when it is perceived as most useful and needed. Moreover, having access to information within an eHealth solution and being able to repeat and confer this information with HCPs were also considered major benefits. Participants who were less positive toward eHealth and digital follow-up care seemed to worry about the “faceless” interaction within an eHealth service and that digitalization would disturb the personal relationship between the patient and HCP. They also feared that valuable information and time would be lost if they were to communicate digitally during an exacerbation. Not being able to manage the various technological aspects of eHealth was also a source of concern, a notion highlighted by several of the participants from all groups.

Overall, the findings from this study suggest that the digitalization of health care provides opportunities and challenges. It seems that patients’ expected benefits of using eHealth might be seen as an important predictor of their willingness to use it. A future eHealth service for patients with HF or patients surgically treated for CRC in the transition from hospital to home could potentially reduce the treatment burden for some as it may support self-management strategies and decrease the number of appointments and personal visits within the health care system. It may also enhance patient’s knowledge and understanding of their condition and provide them with a sense of control. However, to foster patient acceptance, it seems equally important that a future eHealth solution have a human component and focus on becoming a positive contribution to the patients’ daily life and not just on the negative aspects of living with a long-term illness. As this study indicates, some patients may not perceive it as useful to be reminded regularly about having a chronic illness, especially those who already have a social system that provides them with sufficient knowledge and support. Furthermore, some of the time saved by using eHealth and, thus, not having to physically attend health care appointments will be substituted by additional self-monitoring work and other health care tasks [65] such as taking various measurements, answering checklists, or digitally communicating with HCPs. For some patients, this may be perceived as adding to the treatment burden [65].

### Comparison With Prior Work

Patients value education on disease and disease management, specifically information about health status and symptoms, exacerbations, and new challenges [62,66]. “The more you know, the safer you feel” has been expressed by patients with other chronic diseases [67]. Nevertheless, some might struggle with transforming the information they receive during hospitalization into action after discharge. In addition, the health care system today seems to shift a steadily growing list of self-management responsibilities and tasks to the patients’ posthospital discharge, which requires considerable effort from the patients [3]. Hence, patients need self-management support to respond to physical and mental changes and manage their day-to-day challenges and decisions after hospital discharge [13]. Self-management integration is an ongoing process that includes various phases. Seeking effective self-management strategies and creating routines and plans of action are highlighted as 2 crucial steps [14]. However, as the findings of this study demonstrate, many participants described it as challenging to independently seek appropriate strategies and create proper routines in everyday life shortly after hospital discharge. Some lacked a basic understanding of their diagnosis and its symptoms or unexpected complications they should be aware of. Hence, to better meet the supportive care needs of patients with chronic conditions and help them with their self-management tasks, it could be beneficial to provide them with an extended support system through an eHealth service that offers them information, practical advice, and psychosocial support after hospital discharge.

The importance of engaging in self-management activities after discharge and developing more tailored eHealth solutions has been promoted in earlier research [24,31,68]. The term “perceived usefulness” is an important predictor of the acceptance of eHealth, and an eHealth service is more likely to be accepted if the perceived benefits of using the service are outweighed by the negative consequences of having to act on and deal with the disease [63,69]. This study suggests that each patient group had different needs regarding self-management support after hospital discharge. However, the findings also showed common self-management challenges after care transitions across the patient groups. Thus, developing a generic intervention that “fits all” may be possible assuming that the service contains targeted information and functions tailored to fit each diagnosis and self-management support needs. Patients’ perceived benefits of using eHealth could also increase if the service is developed in response to users’ needs rather than as a technological innovation [37]. Moreover, research shows that patients with NCDs want professional support through eHealth services, including human contact to help them address health issues [63]. However, eHealth cannot substitute the personal interaction between patients and HCPs. A nurse-assisted eHealth service will allow patients to communicate their self-management challenges and receive self-management support from a designated NN through a digital service. By designing an eHealth service that considers the holistic needs of patients, clinicians (ie, NNs) can support patients in their transition to self-management [13]. Continuous self-management support from HCPs after hospital discharge could also help patients become more knowledgeable and, at the same time, make them more confident in their skills to manage their illness [60,70]. This could increase patients’ compliance with their health care regimen, which can lead to a reduced number of hospital admissions [71]. As the risk of rehospitalization is high during the first weeks at home (30% for patients with HF [72] and 15% for patients treated for CRC) within the first 30 days after discharge [73], the transition period from hospital to home seems to be an appropriate time to offer digital follow-up care to patients with long-term illnesses such as HF or CRC.

### Strengths and Limitations

This study has several strengths. It included different stakeholders’ views and opinions, which gave varied insights into self-management challenges after hospitalization. Moreover, the study participants varied in gender, age, and educational level, which may have provided this study with a broad perspective on how a nurse-assisted eHealth service could be best designed. Furthermore, in this qualitative study, the aspects of trustworthiness were covered by establishing credibility, dependability, confirmability, and transferability [74]. The credibility and dependability were assured by describing the analytical process in detail and using researcher triangulation throughout the analytical process. Confirmability was assured by presenting the various steps of the analysis, along with a broad overview of data extracts from the participants, in an appendix to make it possible for the reader to agree with and understand the logic of the findings. Transferability was assured by providing the reader with a detailed description of the background and context of the study and focusing on the participants’ stories when presenting the analysis [74].

This study also has some limitations. First, the participants answered questions about an imaginary digital solution. Thus, some of the perspectives and suggestions from the participants will be difficult to transfer and apply within the limits of the future service. Second, the patients in this study answered questions about postdischarge supportive care needs retrospectively, which may have introduced a memory or recall bias [75]. Third, the RNs were recruited for this project because of their interest in eHealth and motivation to provide follow-up care after hospital discharge. Furthermore, the GPs who volunteered to participate may have been more engaged, motivated, and interested in the use of eHealth than the average GP. This may have affected the transferability to both nurses’ and GPs’ perceptions of eHealth in general. Finally, most of the recruited participants who were surgically treated for CRC experienced their discharge period as relatively complication-free. Therefore, their perspectives may not be applicable to the general population of patients with CRC, specifically to patients who experienced complications. Perhaps purposive sampling [52] would have been better suited to capture the diversity within this patient group.

### Conclusions

This study explored stakeholders’ experiences with supportive care needs and their perspectives on eHealth as transitional care for patients with HF and patients surgically treated for CRC as part of an iterative development process of a planned nurse-assisted eHealth service. Both patient populations need specific and tailor-made information on what to expect when transitioning from hospital to home to be as well prepared as possible for self-management tasks. Moreover, they need guidance on how to monitor their health conditions and options for communicating changes to HCPs to avoid uncertainty and anxiety. At the same time, the results indicate that eHealth follow-up services must be adapted according to the severity of the patient’s condition and level of self-management confidence.

This study is valuable as it contributes necessary information from both primary (ie, patients) and secondary (ie, HCP) sources that can ensure the relevant and safe follow-up of patients with NCDs during challenging phases of a care pathway. It suggests eHealth as a possible asset with the potential to bridge the health care void experienced by many patients following a hospital admission. It may fill the resource and knowledge gaps faced by patients with NCDs when performing self-management tasks and prevent unnecessary anxiety and uncertainty among patients. Furthermore, this study stresses the need to tailor the content, functions, and delivery mode of eHealth services to achieve a patient-centered, feasible, and acceptable follow-up after hospitalization. In addition, it may add value to the planning and development of eHealth interventions for other patients with NCDs.

## Appendix

Multimedia Appendix 1 Analysis steps of interviews with patients with heart failure.

Multimedia Appendix 2 Analysis steps of interviews with patients with colorectal cancer.

Multimedia Appendix 3 Analysis steps of nursing interviews.

Multimedia Appendix 4 Analysis steps of general practitioner interviews.

## Abbreviations

CRC: colorectal cancer
GP: general practitioner
HCP: health care professional
HF: heart failure
NCD: noncommunicable disease
NN: nurse navigator
RN: registered nurse
