# Supplementary material for: Designing a Future eHealth Service for Posthospitalization Self-management Support in Long-term Illness: Qualitative Interview Study
Source: JMIR Hum Factors. 2023 Feb 6;10:e39391. doi: 10.2196/39391 (PMC9941902; doi:10.2196/39391)
Supplement: Multimedia Appendix 1 [file humanfactors_v10i1e39391_app1.docx]

**Multimedia Appendix 1: Display of data analysis process – Heart Failure**

| **Main theme** | **Sub theme** | **Code** | **Data extracts** |
| --- | --- | --- | --- |
| Expecting information, reassurance and guidance when using eHealth for HF and CRC self-management | A need for personalized information and advice about what to expect after discharge  A need for personal interaction to reduce post-discharge uncertainty and anxiety | Advice about disease, symptoms, and disease course  Advice about medication, effects, and side-effects  Advice about who to contact and when  Need for emotional support after hospital discharge  Need for practical advice after hospital discharge | If they could have described clearly what was going to happen next. If something was written like: This is how long you can expect things to take [HF 1]  What was really surprising about this, was how long it took before I felt I was back on my feet again. I think it varies from person to person, but for me it was a traumatic experience, and I was very worn out [H5]  What can you do? How much you can pressure yourself? How can you live? Can you live as usual? [HF 7]  My main problem was getting use to all the medications. I mean adjusting….in a sense that you go around dizzy half the day [HF 1]  I would like to know what the different medication does, like beta blockers and such - what does what, and why do I get them? I wanted to know how sick I really was [HF 8]  I didn`t know where to call if I wanted to ask about something. That was the worst – I didn`t know where to call [HF 4]  That someone sent you a message, like: Are you ok? How are you? That would have been very helpful. It doesn`t take more than that [HF 5]  It would have been nice if someone cared. I know I’m just one of many, but if only someone had asked me after a week or so. I would have felt a bit more at ease because then I would have known that someone cared, and to care – that means a lot [HF 4]  When I came home, I had nobody to ask unless I went to my GP, and she couldn`t answer very much about my heart. So, it would have been much better if I had someone to ask, like a peer or something [HF 2]  If you could meet others with similar experiences and ask like: What was your reaction to that? Maybe get some kind of confirmation that your thoughts and feelings were the same as everybody else`s [HF 5]  It could have been ok to ask someone about these little things that pop up – to ask someone right away. I got sick from some of the medication and wondered: What is this? Is it the medicine or is it something else? So, there were lots of things that might have been useful to ask someone about [HF 6]  I wondered about everything. Can I go for a walk? Can I go until I break a sweat? Can I exercise? I feel a pounding, is that normal when I’m sitting down? These common practical things [HF 7]  I wish I could have asked question whenever I thought of something, and got feedback as soon as possible so I didn`t go around and worried so much [HF 3] |
| Expecting eHealth to be comprehensible, supportive and knowledge-promoting | A need for a  manageable and useful eHealth solution  A need for different communication tools and sources for knowledge acquisition | Easy to operate, easy access and distinct layout  Supportive and understandable information  Digital communication with HCP  Gaining knowledge and skills through various functions | The most important factor for me is that it has to resemble other things that I use, so that I don`t have to learn something new. That I can just open it and start using it [HF 1]  That you can enter it easily. It can`t be so advanced that it becomes a strain to use [HF 4]  To get people to use it, it must be as easy as possible. If it were too much fuzz or to many choices, I would have skipped it. I wouldn`t be bothered. I know myself that well [HF 6]  Most importantly there must be a clear button for on and off and maybe a plug for headphones, and maybe a rehearsal or demonstration video the first time you use it… and of course a clear and visual text because it will be mostly used by older people [HF 8]  The information has to be presented so that everyone can understand it [HF 1]  I prefer video. It feels considerably safer when you see someone, and someone sees you [HF 8]  To get answers as fast as possible I would prefer to use chat. Chat is a fast way to get a response [HF 4]  In the beginning I had some difficulties remembering my medication in the evening, so it probably would be nice to have a reminder [HF 6]  Checklists would be most useful for me. Maybe once a week with questions like: Have you had any symptoms? Are you sensing any kind of discomfort? Are you reacting to any of the medication? Then you might detect changes earlier too [HF 5]  Maybe these orientation videos - like what medication does what. That would have helped me [HF 3]  I would have liked to have it (self-monitoring) during the periods I increased my medication dosages, to see how far I could go before I got dizzy. When you see the connection between your condition and how it affects you, you might be more motivated to go for a walk, or not eat that chocolate or [HF 8]  That (self-monitoring) would have been a great thing to have, because then I could have been more attentive, because I tend to be a bit careless about my weight [HF 9]  That you can see from day to day if everything goes in the right direction - if you stagnate or go backwards, and that you can compare your results at the end of the month or week [HF 6]  I like statistic - to see and compare different measurements, so that I can see over time which way it goes [HF 7] |
| Recognizing both advantages and disadvantages of eHealth services for NCD self-management | Recognizing eHealth as a tool for follow-up care  Concerns about eHealth as a tool for follow-up care | Feeling of safety by being monitored at home  Lack of confidence/trust in being monitored at home | If I was offered this (an eHealth service) shortly after discharge, I wouldn`t hesitate, because then I could have been monitored at home, and I would have felt safe. So, there is no doubt I would have accepted. I think it is a good thing to offer [HF 3]  I think everyone with a heart condition would appreciate this. I told my husband: this must be ideal for those with questions. I think I would have been much calmer. It is one of the smartest things you have created [H4]  It could have made me feel safer, and maybe I wouldn`t have felt completely left alone during my most vulnerable period [HF 10]  I weigh myself every morning, and if I was to measure my blood pressure as well, I think I would get stressed [HF 6]  I think that would be a source of stress. I have a drawer full of medicines in in my house, and if I were to have this kind of medical equipment at home as well, it would be too much. So, I would avoid it - having all kinds of devices at home [HF 3] |
