# Supplementary material for: Designing a Future eHealth Service for Posthospitalization Self-management Support in Long-term Illness: Qualitative Interview Study
Source: JMIR Hum Factors. 2023 Feb 6;10:e39391. doi: 10.2196/39391 (PMC9941902; doi:10.2196/39391)
Supplement: Multimedia Appendix 2 [file humanfactors_v10i1e39391_app2.docx]

**Multimedia appendix 2: Display of data analysis process – Colorectal cancer**

| **Main theme** | **Sub theme** | **Code** | **Data extracts** |
| --- | --- | --- | --- |
| Advice about what to expect after surgery  Expecting information, reassurance and guidance when using eHealth for HF and CRC self-management | A need for personalized information and advice about what to expect after discharge  A need for personal interaction to reduce post-discharge uncertainty and anxiety | Advice about what to expect after surgery  Advice about who to contact and when  Need for emotional support after hospital discharge  Need for practical advice after hospital discharge | If I just could have gotten answers to the most common questions that arises after such surgery [CRC 1]  They did cut out a piece of my bowel, right? And had a lot of gas in my stomach. Is that normal? [CRC 3]  If I could have asked someone about my stomach. Is going to the toilet seven times a day normal? That sort of thing [CRC 2]  I had a problem with my surgical wound - a leakage. I had to change the dressings two to three times a day and I was worried it would get infected. That was a problem [CRC 3]  Information about how long the pain would last. For me, it lasted for months. Then maybe you don`t feel abnormal when you experience such pain from your rear end [CRC 9]  In the beginning I had some trouble knowing what to eat. Can I eat that? Is it dangerous? Is it caustic? Is it too heavy for my stomach? [CRC 7]  I went to my GP, because that is what you are told to do if something happens. So, I did, and I got the information I needed from him [CRC 3]  It is the waiting period and the uncertainty that is the worst. That you don`t know. That bothered me a lot. Would I need radiation? Could I get the treatment here? [CRC 7]  If I had a way I could have conveyed my questions to the right person and that someone answered me [CRC 5]  I would have liked to have asked some questions to someone who knows. That we could have communicated a bit back and forth [CRC 1] |
| Expecting eHealth to be comprehensible, supportive and knowledge-promoting | A need for a  manageable and useful eHealth solution  A need for different communication tools and sources for knowledge acquisition | Easy to operate, easy access and distinct layout  Reasonable response time  Supportive and understandable information  Digital communication with HCP  Gaining knowledge and skills through various functions | Easy to use and that you found what you were looking for. Clear and distinct. It can`t be too complicated [CRC 2]  There has to be someone in the other end who will answer you fairy quickly if you have questions that needs to be answered [CRC 9]  That you become calm after reading the information. No words of panic, more like reassuring and calming information [CRC 4]  If the information could be adapted to us without any formal education. That the information could point us in the right direction so to speak [CRC 8]  I think chat is very good. I have used it in other contexts where I have wondered about things or I have needed help, and I think chat is very functional [CRC 4]  I would prefer video. I would want them to see the surgical wound. To see if I needed to change the dressing, or if I needed to go to the hospital and do something about it [CRC 3]  I would use both. Chat would be fine, but facetime wouldn`t be wrong either [CRC 9]  I think checklists are great. Say you have to change your bandages, or other things that needs to be done regularly [CRC 8]  I was bit anemic, so I would have liked to be reminded about things like that maybe. Like: Call your doctor and check your blood percentage. Those things are important and needs to be kept an eye on [CRC 8]  If you google colorectal cancer, you get these illustrations. So that could be useful – to see pictures of what they are doing during the surgery [CRC 3]  At least there should be some information or introductions and short pieces of film. That wouldn`t be so stupid, especially considering people with bad hearing or vision [CRC 5]  I imagine that some of the questions from patients are recurrent. So if they could be made more visible - common information and answers [CRC 5] |
| Recognizing both advantages and disadvantages of eHealth services for NCD self-management | Recognizing eHealth as a tool for follow-up care  Concerns about eHealth as a tool for follow-up care | Feeling of safety by being monitored at home  Lack of confidence/trust in being monitored at home | My first question is: What is this(eHealth) good for? Well, I think it`s psychological. It would seem like someone cared about you, right? [CRC 1]  When everything doesn`t go as planned it could be important to have an app to help people afterwards. When something was wrong with the wounds, or…some might collapse completely and might have needed to contact someone [CRC 2]  Of course it would be beneficial to have access and have a dialogue with experts…to have an app and ask about things you weren`t familiar with or insecure about [CRC 3]  Maybe if I relapsed or something, but as long as I feel well. If I were to start reading information in an eHealth solution, I suddenly would have felt all the symptoms as well [CRC 6]  I had my discharge letter, so I don`t know. I didn`t feel so sick that I needed it, but maybe if I was differently sick [CRC 5]  We are all very different, but if everything goes well, you don`t have the same needs as you would have when things don`t go so well [C2]  I wouldn`t need it, but if things were different. Those who get a stoma, they might say more about what they needed. I didn`t have any problems which made me feel I needed to come in contact with anyone [CRC 7] |
