# Supplementary material for: Designing a Future eHealth Service for Posthospitalization Self-management Support in Long-term Illness: Qualitative Interview Study
Source: JMIR Hum Factors. 2023 Feb 6;10:e39391. doi: 10.2196/39391 (PMC9941902; doi:10.2196/39391)
Supplement: Multimedia Appendix 3 [file humanfactors_v10i1e39391_app3.docx]

**Multimedia appendix 3: Display of data analysis process - Nurses**

| **Main theme** | **Sub theme** | **Code** | **Data extracts** |
| --- | --- | --- | --- |
| Expecting information, reassurance and guidance when using eHealth for HF and CRC self-management | A need for personalized information and advice about what to expect after discharge  A need for personal interaction to reduce post-discharge uncertainty and anxiety | Advice about what to expect after surgery  Advice about disease, symptoms, and disease course  Advice about medication, effects, and side-effects  Advice about who to contact and when  Need for support after hospital discharge  Need for practical advice after hospital discharge | Many worry about their stomach, what`s normal, what will happen when they get home. There`s diarrhea or constipation, and there`s the pain - that`s typically recurring questions [CRC Nurse 2]  Things that has to do with bowel function, when will that be normal? Because that usually takes a while after surgery. Then there`s the surgical wound, the stitches, and the lifting. When can they start doing things like that again? [CRC Nurse 1]  They have to remember to eat even though their sense of hunger haven`t returned. Some are nauseas or have a reduced appetite, and some have a reduced stomach and can`t manage to eat as much as before. But they have to get enough nutrition [CRC Nurse 1]  I`m under the impression that they don`t understand the mechanisms behind heart failure, so when something happens, they don`t manage to connect that it`s actually related to their disease [HF Nurse 3]  What can I expect to manage? And also: Am I able to go for a walk? Walk the stairs? Play with my grandchildren? How hard can I push myself? What is ok? [HF Nurse 5]  The biggest challenge for HF patients is the amount of information. There`s so much they need to know and difficult for them to know what`s most important. The information tend to slip away because it`s too much [HF Nurse 3]  They need information about their medication, what they are taking and why, and also a bit about the side-effects. We should also make a point of not suddenly stopping their medication if they are experiencing side-effects, but rather talking to someone about reducing the dosage or changing the brand [HF Nurse 6]  Information on what kind of follow-up they are going to get, who they should contact when they have questions and who is responsible for their follow them up. Is it their GP? Is it the hospital? And if they need help more acute? Emergency room or 911 [HF Nurse 6]    When they get home and everything is over. They`re diagnosed, examined, operated and back home within two weeks. It`s like: WOW – what just happened? I had cancer – can I relapse? Patients aren`t able to think before their discharged. This fast-track thing is great, but psychological everything is going too fast [CRC Nurse 1]  Those who are hospitalized, and has had heart failure for some time, they understand very well what is happening. They are often tired and maybe also depressed and anxious. They`ve lived with the disease for a while and manages less and less and that is tiresome [HF Nurse 5]  They feel like they are given a lot of responsibility in following up on their disease. Scheduling appointments with their GP, going in for check-ups, follow up on changes in medication and then they`re told to pay attention to their weight, edemas, breathing and all of that - adjusting their life to the diagnosis. So yes, I do think patients’ sense that responsibility [HF Nurse 4]  They wonder about so many different things, so to know that they are not alone with a stoma - that there is someone they can contact, like peers that have gone through the same thing” (CRC Nurse 2)  They`re the ones that`s had the most life-changing operation, so even though it`s just for a few months – many just aren`t capable of relating to the stoma even though it`s only there for a short period of time [CRC Nurse 1] |
| Expecting eHealth to be comprehensible, supportive and knowledge-promoting | A need for a  manageable and useful eHealth solution  A need for different communication tools and sources for knowledge acquisition | Easy to operate, easy access and distinct layout  Supportive and understandable information  Digital communication with HCP  Gaining knowledge and skills through various functions | It has to be very user-friendly. My 75-year-old grandmother can easily use an iPad and Facebook, but I feel there are great variations in that age group on how familiar they are with using technology, so it has to be easy to use - simple, plain, and harmless [HF Nurse 4]  You shouldn`t overload them with information. So, as easy as possible, simplify complicated things and also a clear and straight forward language [HF Nurse 6]  When it comes to medication it may be too much if you go into details and use professional language. The information has to be written in a way that normal people can understand, or else I think it creates more uncertainty [CRC Nurse 2]  I would like that the patients could make me a memo, which then will be made available to me so that I can see what they have written and then talk to them about these things [CRC Nurse 1]  I think it would be easier to write instead of calling. I don`t need to see them for them to tell me they are constipated or have trouble eating [CRC Nurse 2]  That they can write questions back and forth. Simple, concrete questions about their daily form or medications. A question-and-answer function would be good [HF Nurse 5]  I think video would be optimal because you can see the patient and also respond to their facial expressions. And you can really see the effect of asking them questions or give them information [HF Nurse 3]  Short questionnaires like: How`s your stool? Do you have pain? Easy questions to check if the patient manages to do what he is supposed to do at home [CRC Nurse 1]  I think pictures gives a better impression. It`s easier to understand and easier to remember. Pictures of the heart maybe, how it pumps, or a pacemaker – how that works [HF Nurse 5]  That there are links to reliable sources on the internet. That would be helpful [CRC Nurse 1]  Maybe they could weigh themselves to see if they manage to maintain their weight, because some lose their appetite after the surgery, but they still get discharged [CRC Nurse 2]  If they weigh themselves every morning and plot it into the system. I think that would be reassuring for them - that they can get feedback on whether their measurements are ok or not. And also, they become familiar with normal measurements, and also what is normal for them (HF Nurse 5) |
| Recognizing both advantages and disadvantages of eHealth services for NCD self-management | Recognizing eHealth as a tool for follow-up care | Feeling of safety by being monitored at me | I think it could give the patients an enormous feeling of security. They seem to relate to different measurements fairly quickly, so I think it would be reassuring [HF Nurse 6]  I think this kind of eHealth service first and foremost will provide good follow-up care. During hospitalization the patients have the opportunity to ask questions round the clock. This stands in sharp contrast to being discharged and not having any contact with the hospital [CRC Nurse 1]  eHealth may contribute to patients actually getting the information they need. And because they are supported by us, I think it may help the patients manage their own disease. We can support them and because the information is adapted it will give them increased secureness, which also may effect readmissions [HF Nurse 3]  I think digital follow-up will be better for the patient because they are often sent home with many unanswered questions, and often next of kin have questions too [CRC Nurse 2]  I think you could detect breathlessness sooner, and then they can act on it at an earlier stage. That might contribute to the patients being less anxious, and more capable of managing everything at home [HF Nurse 4] |
