# Supplementary material for: Designing a Future eHealth Service for Posthospitalization Self-management Support in Long-term Illness: Qualitative Interview Study
Source: JMIR Hum Factors. 2023 Feb 6;10:e39391. doi: 10.2196/39391 (PMC9941902; doi:10.2196/39391)
Supplement: Multimedia Appendix 4 [file humanfactors_v10i1e39391_app4.docx]

**Multimedia appendix 4: Display of data analysis process – General Practitioners**

| **Main theme** | **Sub theme** | **Preliminary theme** | **Data extracts** |
| --- | --- | --- | --- |
| Expecting information, reassurance and guidance when using eHealth for HF and CRC self-management | A need for personalized information and advice about what to expect after discharge  A need for personal interaction to reduce post-discharge uncertainty and anxiety | Advice about what to expect after surgery  Advice about disease, symptoms, and disease course  Advice about medication, effects, and side-effects  Advice about who to contact and when  Need for support after hospital discharge  Need for practical advice after hospital discharge | After hospital discharge, issues such as complications are in focus. Infection or not infection, what`s normal and what`s not normal, how much leakage is normal - patients don`t know these things [GP 1]  I feel that they mostly struggle with their bowel, whether it`s constipation, diarrhea, or gas. It may take up to 3 months before they have control, and I think that they might benefit from a bit of guidance [GP 3]  Many of the CRC patients struggle with lethargy and fatigue, and they are not fully prepared how much of a bother this will be in their life. That they will be living a bit on the minus side [GP 8]  The importance of restitution. You often see that in the colorectal cancer patients. They often say: I have to take a nap 3 times a day – I have to rest because my strength has not returned yet [GP 6)  Many patient don`t familiarize themselves with their condition and lack engagement about their treatment - how everything works and what they should be paying attention to. If you take a random selection of heart failure patient and ask them if they weigh themselves, many will answer: No I don`t. Why should I do that. And many knows very little about what might worsen the condition. So, there are some real knowledge gaps here [GP 13]  Those with heart failure often visit me to get some sort of sum up of the road ahead - a future plan for follow up. And they also feel breathless and we discuss their blood pressure and medication – if it`s working or if they`re dizzy. Things like that [GP 11]  I think HF patients need information about the accompanying symptoms that they don`t always manage to connect to their disease. Like some of the psychological reactions that they can get [GP 7]  I think it is important that they know why they need medication, what the medication does for them and that they can`t stop taking them, because that`s often an issue. They don`t know why it`s so dangerous for them to skip their betablocker a week or two. And some take extra diuretics without coming in for checkups, and get electrolyte disturbances [GP 9]  They should be informed about what they can expect, and also clear information about when they should contact someone [GP 3]  That they get information about what is normal, and if you get symptoms like this you need to contact someone [GP 11]  I think the most important thing is that they get concrete information about which symptoms should lead to medical attention, and which symptoms need acute medical attention. [GP 8]  The heart has a special place in our psyche, so when something happens to the heart, they get many questions. They often have an existential fear that the heart will just suddenly stop [GP 4]  Many have psychological challenges like: I`ve had cancer and I know people die of cancer, so what about me? I had Dukes stadium this and that, what does that mean? So, of course it`s a huge mental strain [GP 5]  As soon as they can pee and eat, they are assessed as discharge ready, but when they get home it is not really ok after all. Often it doesn`t work that well at home [GP 2]  They wonder how they are doing, like: Why do I have this? Will I die of heart failure? It really is a horrible diagnosis because it sounds like everything is failing, and purely medical speaking, it also does. And also: How long will I live? For how long will my heart last? [GP 5]  If they visit their GP shortly after discharge, it`s almost always because something is unclear or something has come up and they are uncertain whether it`s related to the treatment they got during hospitalization [GP 13]  I think both activity and diet is important to follow-up on. To provide them with a sense of security that they can accomplish something on their own with the help of activities and a healthy diet [GP 7] |
| Expecting eHealth to be comprehensible, supportive and knowledge-promoting | A need for a  manageable and useful eHealth solution  A need for different communication tools and sources for knowledge acquisition | Easy to operate, easy access and distinct layout  Supportive and understandable information  Digital communication with HCP  Gaining knowledge and skills through various functions | The fact that it is digital is already a barrier for many, so it has to be an easy interface most people can operate [GP 1]  It can`t have an advanced interface so they get lost or afraid to push the buttons. It must be safe and easy to deal with, and easy to register the measurements [GP 2]  To have access to information in a language they understand. That could also be beneficial for immigrants. You actually have a unique opportunity to help a future group of patients [GP 4]  They often need confirmation on the information they`ve received in the hospital, because everything hasn`t sunk in. Many are in a state of crisis when they`re in the hospital, and often need some kind of verification or explanation after they come home [GP 5]  That there has to be an easy way for the patient to get in contact with a living human being in the other end if they have questions and that they can call or chat… or face to face. Many will find it easier to relate to a face then a text [GP 6]  In praxis I think a chat-service would be more convenient to operate than a video. If you have both, it has to be the ones operating from the hospital that has to initiate the video [GP 4]  It would be very interesting to get an overview on patients` activity patterns, heart rate, blood pressure, weight. And also for the patient – a kind of diary on their measurements [GP 8]  The patients can try getting back to doing activities again. How did that go? Maybe it will feel safe for them to get started again [GP 11]  You could ask the patient to do some kind of self-evaluation once a week and then give them feedback on the development and motivate them to stay physical active [GP 9]  They could register their activity, or to monitor how much they eat or if they have a good sleeping rhythm. That sort of things. I think that would be very suitable [GP 7]  They should be able to read about different associations they can join, local clubs. [GP 11] |
| Recognizing both advantages and disadvantages of eHealth services for NCD self-management | Recognizing eHealth as a tool for follow-up care  Concerns about eHealth as a tool for follow-up care | Feeling of safety by being monitored at home  Lack of confidence/trust in being monitored at home | I think they would benefit a lot from a type of follow-up outside the GPs office. They could really benefit from motivation and assurance from a nurse through such a solution [GP 7]  The key is accessibility to another human being. You can have a dialogue without feeling that it`s unnecessary or that you put a strain on the health care system [GP 10]  I think it`s important that you don`t put too much medical stuff into digital services, but create a platform where you can give information which matches the patient - to help them cope [GP 2]  I think eHealth is a good way to reach people when they need help, but I don`t think it`s wise to let it replace the GPs evaluations [GP 9]  We see now during the pandemic how important it is to see the patients. How much information you get by actually seeing the patient - how they walk into the office, how they breathe. So, I don`t think you should eliminate GP consultations too much [GP 3]  There are so many platforms and clinics that are being developed and I think the patients are getting a bit confused, so I am worried about… well, about the complexity of it all [GP 12] |
